# Unusual Anterior Mediastinal Tumors Treated at a Tertiary Thoracic Center: A Case Series Analysis

**DOI:** 10.7759/cureus.17625

**Published:** 2021-08-31

**Authors:** Ambrish Kumar, Shailendra Kumar, Jitendra Kushwaha, Vaibhav Raj, Archana Mishra

**Affiliations:** 1 Department of Vascular Surgery, King George’s Medical University, Lucknow, IND; 2 Department of Thoracic Surgery, King George’s Medical University, Lucknow, IND; 3 Department of General Surgery, King George’s Medical University, Lucknow, IND

**Keywords:** chondroid hamartoma, thymic carcinomas, fibrosarcoma, teratomas, mediastinal tumors

## Abstract

Several tumors arise from different structures within the mediastinum. Although each type of mediastinal tumor has a predilection for a specific compartment, the progression of growth from one compartment to another can occur. The anterior mediastinum is the site of several tumors that pose interesting diagnostic and therapeutic challenges to thoracic surgeons. The anterior mediastinum is the seat of the majority of neoplastic growths within the mediastinum. Thymomas and lymphomas are the most common pathologies of the anterior mediastinum. Tumors of mesenchymal origin (hemangioma, lymphangioma, lipomas) and their malignant counterparts may occur in any of the mediastinal compartments. Less common tumors of the anterior mediastinal compartment are ectopic thyroid and parathyroid tumors, germ cell tumors, mesenchymal origin tumors, hemangiomas, and cervicomediastinal hygromas. Most of the mediastinal growths usually remain clinically silent until they become large and cause compressive symptoms. Here, we present a case series of five anterior mediastinal tumors consisting of solitary benign teratoma, fibrous benign tumor, malignant fibrosarcoma, hamartomatous chondroma, and malignant thymoma.

## Introduction

The mediastinum is the space within the chest located between two pleural cavities. It extends from the thoracic inlet to the superior surface of the diaphragm. Several organs such as the heart, great vessels, trachea and bronchi, esophagus, thymus, and lymphatic vessels are contained within the mediastinum. Growths of the middle mediastinum commonly comprise congenital cysts and posterior compartment neurogenic tumors. More commonly, some tumors originate from the anterior mediastinum. Most of the anterior mediastinum masses are either thymomas or lymphomas with incidences of 35% and 25%, respectively. Some unusual tumors of anterior mediastinum need special mention as they are of clinical managerial significance. Germ cell tumors (GCTs) arising in the mediastinum comprise only 3% of all overall GCTs. Of the thymic masses, less than 1% of lesions comprised adult malignant thymomas. Mesenchymal tumor of mediastinum comprises only 2% of the neoplasm of the mediastinum, making them very rare [1]. Only cases and small case series of mesenchymal origin tumors have been reported to date. Pulmonary hamartomas are benign neoplasms that comprise mainly of chondroid or chondromyxoid tissues. They comprise 4% to 7% of all solitary pulmonary nodules [1]. These unusual tumors encountered in clinical practice need to be properly characterized and treated. Therefore, here, we discuss five unusual cases of anterior mediastinal tumors.

## Case presentation

Case 1

**Figure 1:**
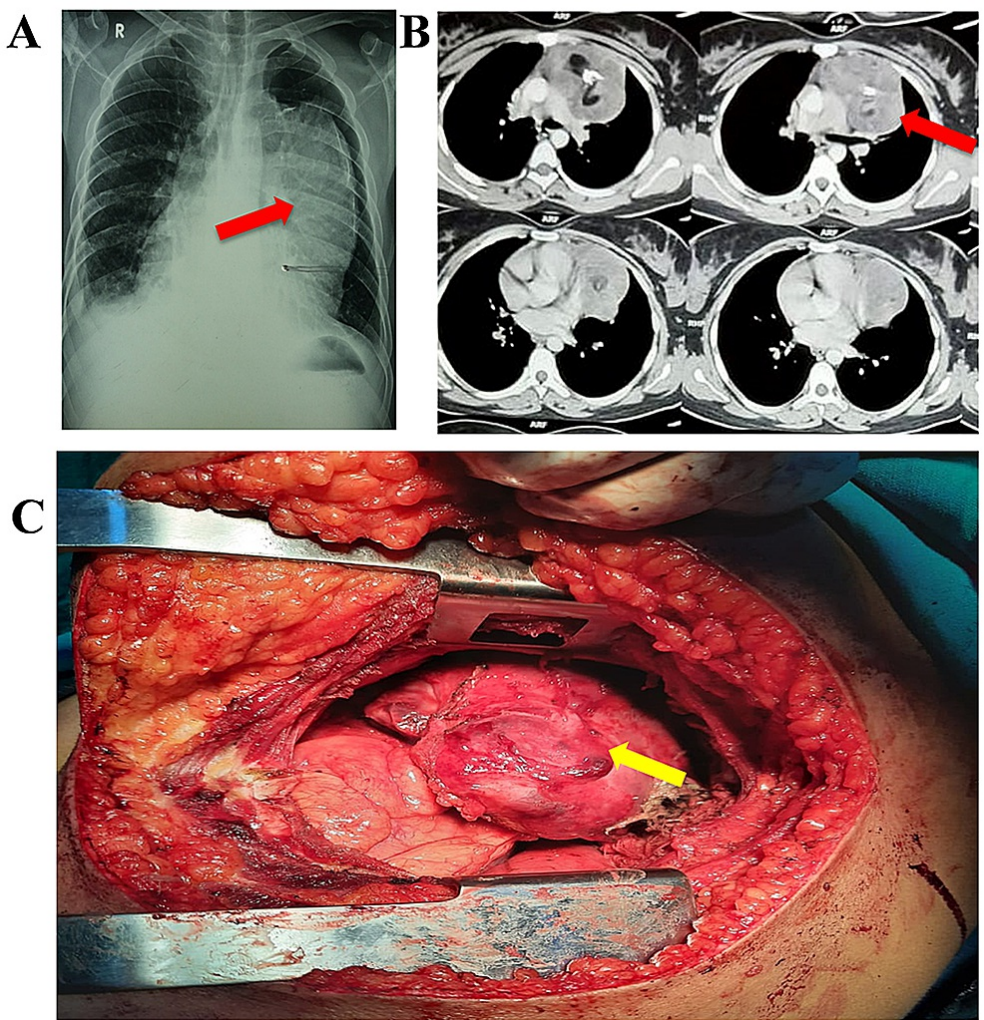
(A) Chest X-ray PA view; (B) contrast-enhanced CT of the chest; (C) Intraoperative picture. Red and yellow arrows represent benign teratoma in radiological and intraoperative figures, respectively. PA: posteroanterior; CT: computed tomography

A 28-year-old female presented with episodes of cough and chest pain for six months. She had no history of hemoptysis but complained of heaviness over the upper chest. She was a nonsmoker, nonalcoholic, and had no concurrent medical illnesses. On examination, she was mildly anemic and otherwise normal. There was no cervical or peripheral lymphadenopathy. Respiratory and cardiac examinations were within normal limits. Chest X-ray posteroanterior view (Figure 1A) revealed a large opacity over the upper chest more on the left side. A contrast-enhanced CT scan of the chest (Figure 1B) showed a large smooth margin lesion in the anterior mediastinum with areas of calcification and necrosis. Based on CT findings, a preoperative diagnosis of benign teratoma was made, and she was planned for left-sided thoracotomy. On exploration, the mass was well defined and capsulated but adherent to the left brachiocephalic vein and many unnamed vessels and close to the aortic arch (Figure 1C). As expected, the final histopathology was benign teratoma (dermoid cyst).

Case 2

**Figure 2:**
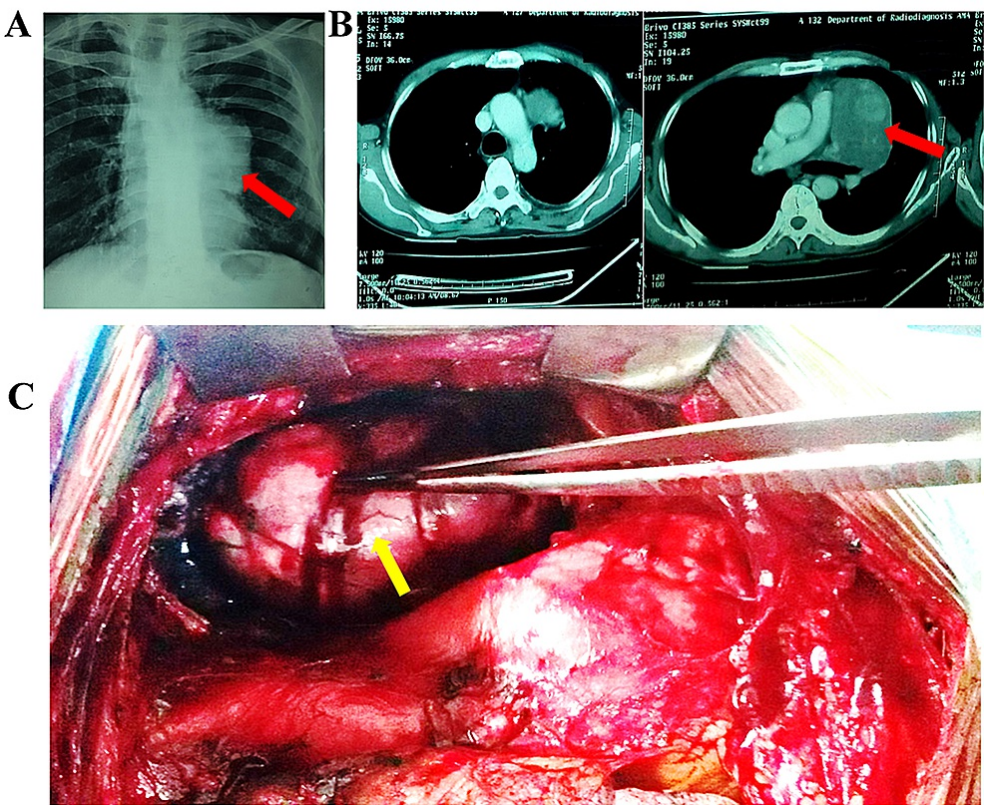
(A) Chest X-ray; (B) contrast-enhanced CT scan; C: intraoperative picture. Red and yellow arrows represent benign fibrous tumor in radiological intraoperative figures, respectively. CT: computed tomography

A 42-year-old male presented with chest discomfort in the form of heaviness over the upper part of the chest for five months. There was no history of cough, shortness of breath, hemoptysis, or fever, suggestive of any chronic inflammatory or malignant neoplastic pathology. Examination revealed slightly decreased breath sounds on the left side with a dull percussion note without diminution of breath sounds over the left upper chest. Chest X-ray (Figure 2A) showed a radio-opaque left-sided mediastinal shadow. A contrast-enhanced CT scan of the thorax (Figure 2B) suggested an anterior mediastinal mass with smooth margins and few signs of calcification. Thoracotomy was planned and the whole mass was excised which was lying over the aortic arch and the main pulmonary artery (Figure 2C). Histopathology confirmed the diagnosis of an anterior mediastinal benign fibrous tumor.

Case 3

**Figure 3:**
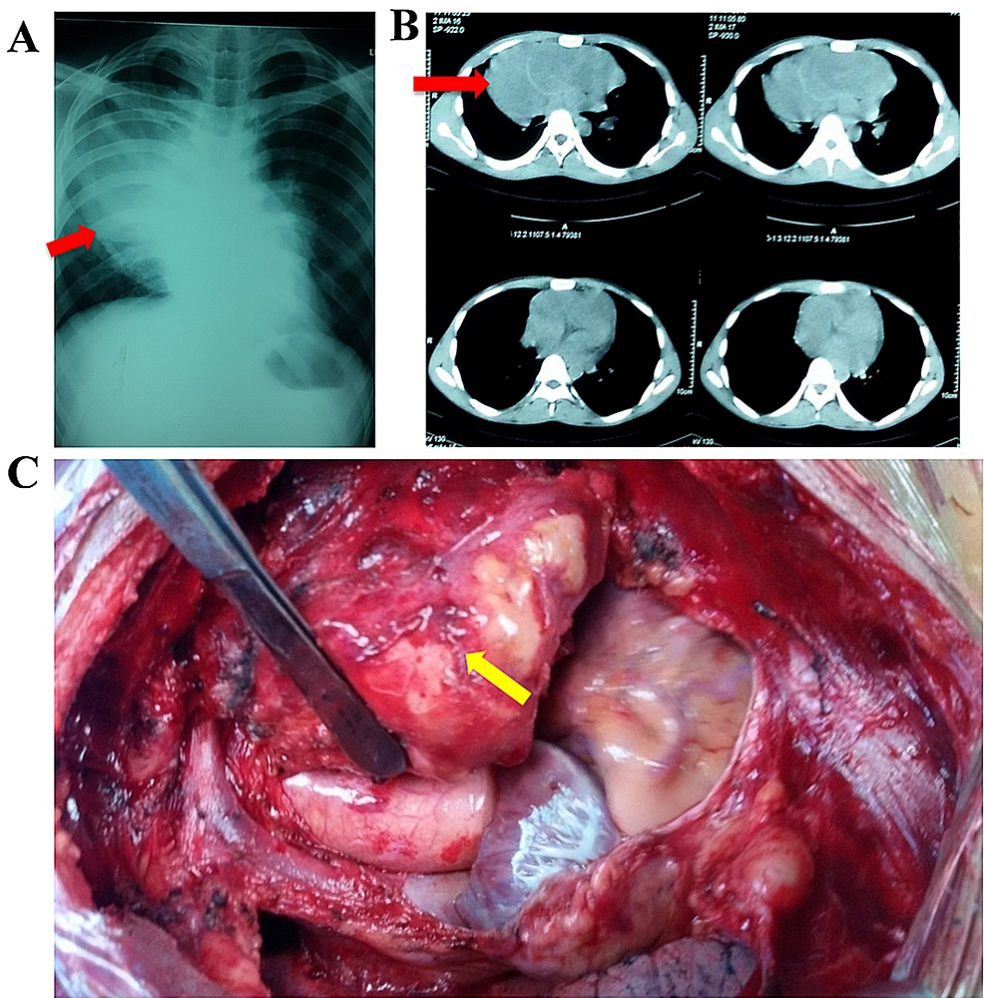
(A) Chest X-ray; (B) contrast-enhanced CT scan; (C) intraoperative picture. Red and yellow arrows represent malignant fibrosarcoma in radiological and intraoperative figures, respectively. CT: computed tomography

A 46-year-old male presented to our outpatient department with a history of chest pain and discomfort on the right upper chest. On examination, we noted signs of venous congestion leading to venous fullness and tortuosity over the neck and head. Chest X-ray (Figure 3A) was suggestive of a large mass in the anterior mediastinum largely on the right side. A contrast-enhanced CT scan (Figure 3B) confirmed a soft-tissue large mass suspicious of neoplastic pathology. Thoracotomy was planned and the mass was excised with an adequate margin of 2-3 cm except for the bases and taking out part of the pericardium. The mass was close to the superior vena cava, right atrium, aortic arch, and main pulmonary vessel (Figure 3C). Histopathology confirmed the diagnosis of malignant fibrosarcoma.

Case 4

**Figure 4:**
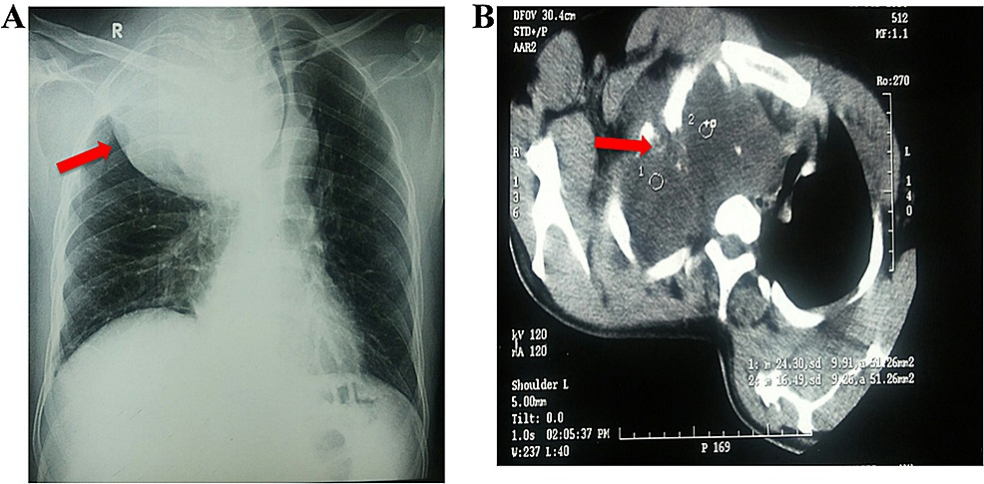
(A) Chest X-ray; (B) contrast-enhanced CT scan. Red arrows represent chondroid hamartoma in radiological figures. CT: computed tomography

A 48-year-old male presented with shortness of breath and slight bulging over the right upper anterior chest wall and supraclavicular fullness for the last two years. He had an episodic dry cough but no history of hemoptysis or chest pain. There was no history of difficulty in swallowing. He was a nonalcoholic and nonsmoker. There was no lymphadenopathy over the neck or at other sites. He developed slight facial puffiness during the last three months. Breath sounds were markedly decreased on the right side and chest movement was restricted over the upper chest. No signs suggestive of Horner’s syndrome were present. Chest X-ray depicted a large radiopaque (Figure 4A) well-defined shadow in the right upper lung field with marked left-sided tracheal deviation. A contrast-enhanced CT scan (Figure 4B) revealed a solid well-defined radiodense mass suggestive of a cartilaginous tumor. CT-guided biopsy from the lesion reported chondroma, after which he was planned for thoracotomy and excision. Intraoperatively, the mass was extending up to the supraclavicular region but had maintained planes with the intrathoracic structures. Adequate debulking was performed. Histopathology revealed chondroid hamartoma which did not require further treatment.

Case 5

**Figure 5:**
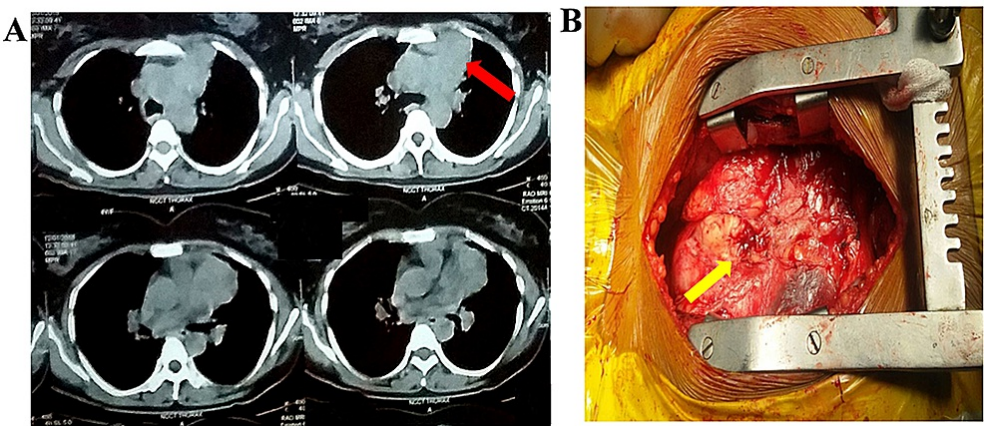
(A) Contrast-enhanced CT scan; (B) intraoperative picture. Red and yellow arrows represent thymic carcinoma in radiological and intraoperative figures, respectively. CT: computed tomography

A 56-year-old male patient came to the department of thoracic surgery with a history of malignant thymoma (Figure 5A) after undergoing chemotherapy at another hospital. He was referred for surgical removal to our thoracic surgery department. Surgical resection (Figure 5B) was done through a midline sternotomy. Histopathology showed undifferentiated aggressive thymic carcinoma. He was given adjuvant chemotherapy after surgery. He died of metastatic complications after three months.

## Discussion

Mediastinal masses include a wide array of diagnoses ranging from benign to highly malignant pathologies. Thymoma is the most common anterior mediastinal mass followed by lymphomas [2]. Primary lymphomas comprise 10% of all mediastinal tumors. The rest of the lesions include goiter, parathyroid adenomas, and GCTs. Benign teratoma is the most common mediastinal GCT.

As mediastinal masses usually present with nonspecific symptoms, a proper investigative workup is required to categorize them according to their location and radiographic nature. A chest X-ray is a routine investigation for any chest symptom; however, it is not sensitive enough to pick up the pathologies, especially when they are smaller in size. Hence, a thoracic CT scan and at times magnetic resonance imaging are required for detailed characterization and anatomic localization of the mass and its relation to the surrounding structures [3].

Anterior mediastinum is the most common extragonadal site for teratomas [4]. They mostly present in the younger age group of 20-40 years, though pediatric cases have also been reported [5]. Benign/mature teratomas have well-differentiated tissues of all embryonic origins, whereas malignant immature teratomas are composed of neuroectodermal elements. They may either present with nonspecific and compressive symptoms or can be detected incidentally. Rare complications include pericardial effusion [6] and rupture into lungs and bronchus, which can lead to trichoptysis and hemoptysis [7]. Similarly, teratoma with malignant transformation has also been reported [8]. Surgical excision is the treatment for mature cystic teratoma with an excellent prognosis [9].

Intrathoracic fibromas are mesenchymal neoplasms that are exceedingly uncommon. There has been persistent confusion regarding the nomenclature of this tumor, which consisted of pleural fibroma, benign mesothelioma, submesothelial fibroma, etc. At present, these tumors are designated as solitary fibrous tumors [10]. CD34 is the most important marker [11]. The majority of them arise from the pleura but can be found in the liver, pancreas, kidney, orbit, and mediastinum [12]. Malignant fibrosarcoma is a comparatively rare entity in the anterior mediastinum. Surgical excision is the accepted treatment and recurrence and rapid metastasis have been reported [13].

Hamartoma is defined as an excess of normal tissue elements at an abnormal site. Though chondroid hamartoma is the most common benign pulmonary neoplasm, its occurrence in anterior and posterior [14] mediastinum has been reported very rarely. They are mostly small in size, peripherally located, found in the fifth and sixth decades of life, and have a male gender predilection [15]. Saadi et al. reported a multicystic pulmonary hamartoma in a 10-year-old boy presenting with a pneumothorax who was successfully treated with surgical excision [16]. Histologically, they comprise adipose, fibrous, and chondromyxoid elements. Chromosomal studies reveal mutations in 6p21 and 12q13-15 and have been classified as benign mesenchymal neoplasms [17].

Thymomas are indolent tumors arising from thymic tissue; however, thymic carcinomas are quite aggressive cancers having a poor prognosis. They are of epithelial origin and have cellular and architectural components of malignancy. The most common subtype is squamous cell carcinoma that may develop de novo or in a preexisting thymoma. The poorly differentiated squamous cell carcinoma can be very aggressive and are locally infiltrative and metastatic. CD5, CD117, FOXN1, and CD205 can be used to differentiate it from other cancers [18]. The International Thymic Malignancy Interest Group database describes the squamous cell subtype as constituting 79% of thymic cancers followed by lymphoepithelioma-like tumors (6%) [19]. Treatment is surgical excision either by open thoracotomy or minimally invasive procedures consisting of video-assisted thoracoscopic surgery (VATS) and robotic surgery.

## Conclusions

We experienced a wide range of anterior mediastinal masses including malignant ones in terms of presentation, diagnosis, and management. Mediastinal masses can be treated very effectively by various surgical approaches including VATS, thoracotomy, or median sternotomy, depending upon their location, size, and surgical expertise. The postoperative outcome is usually excellent in most cases. Malignant entities require a more sophisticated approach based on histopathology to excise the masses with proper margin.
